# Establishment of RT-RPA-Cas12a assay for rapid and sensitive detection of human rhinovirus B

**DOI:** 10.1186/s12866-023-03096-1

**Published:** 2023-11-11

**Authors:** Yongdong Li, Xuefei Wang, Rong Xu, Ting Wang, Dandan Zhang, Weidong Qian

**Affiliations:** 1 Ningbo Key Laboratory of Virus Research, Ningbo Municipal Center for Disease Control and Prevention, Ningbo, 315010 P. R. China; 2 School of Food and Biological Engineering, Shaanxi University of Science and Technology, Xi’an, 710021 P. R. China

**Keywords:** Human rhinovirus B, CRISPR-Cas12a, Reverse-transcription recombinase polymerase amplification, Detection, On-site diagnosis

## Abstract

Human rhinovirus B (HRV-B) is a major human viral pathogen that can be responsible for various kinds of infections. Due to the health risks associated with HRV-B, it is therefore crucial to explore a rapid, specific, and sensitive method for surveillance. Herein, we exploited a novel detection method for HRV-B by combining reverse-transcription recombinase polymerase amplification (RT-RPA) of nucleic acids isothermal amplification and the trans-cleavage activity of Cas12a. Our RT-RPA-Cas12a-based fluorescent assay can be completed within 35–45 min and obtain a lower detection threshold to 0.5 copies/µL of target RNA. Meanwhile, crRNA sequences without a specific protospacer adjacent motif can effectively activate the trans-cleavage activity of Cas12a. Moreover, our RT-RPA-Cas12a-based fluorescent method was examined using 30 clinical samples, and exhibited high accuracy with positive and negative predictive agreement of 90% and 100%, respectively. Taken together, a novel promising, rapid and effective RT-RPA-Cas12a-based detection method was explored and shows promising potential for on-site HRV-B infection in resource-limited settings.

## Introduction

Human rhinoviruses (HRVs) belong to the enterovirus genus in the Picornaviridae family, and are one of the important human respiratory pathogens [1]. HRVs are a major cause of both upper respiratory tract infections (URTI) [2] and lower respiratory tract infections (LRTI) [1], with several clinical manifestation including common colds [2–4], otitismedia, and sinusitis [4–6]. HRVs cause infection in all parts of the world, accompanied by a seasonal pattern. Nowadays, severe clinical presentations caused by HRVs have been frequently reported among specific population, particularly in infants and older individuals with underlying diseases or in moderately or severely immunocompromised patients [7], posing a substantial economic burden for society [8]. Notably, HRVs are the second virus associated with bronchiolitis and pneumonia in high-risk children, and HRV bronchiolitis has been linked with a higher risk level of recurrent wheezing episode or asthma [9]. Moreover, HRV infections impose a substantial economic burden per year due to medical costs and indirect costs from work-related absenteeism [10]. In this context, exploring therapeutic targets and interventional strategies for HRVs are essential to minimize added socioeconomic problems for societies due to HRV infections.

Simlar to other Picornvariueses, HRVs are positive-sense, single-stranded, and non-enveloped RNA viruses [8]. Its genome comprises approximately 7200 nucleotides in size. The genome is composed of a single gene, which is translated into a single polyprotein. After translation, the polyprotein is co- and post-translationally processed into separate proteins via viral and celluar virus-encoded proteinases, including VP4, VP2, VP3 and VP1 and nonstructural proteins (2A, 2B, 2C, 3A, 3B, 3C and 3D) [4]. The genetic diversity of HRV is determined mainly by the external structural proteins VP1, VP2, and VP3, while VP4, aligning itself on the inner side of the capsid, is more conserved in sequence with lower variability. To date, HRVs are mainly subdivided into three species, such as HRV-A, HRV-B and HRV-C [11, 12], based on the 5′-untranslated region (5′-UTR) or the VP4/VP2 region [13]. Unfortunately, there are currently no specific antivirals for HRVs, and vaccination against HRVs is not effective due to over 150 antigenically distinct serotypes of HRV [14].

In view of the rapid rise in cases of infections caused by HRVs in individuals, it has become imperative to exploit rapid and more approachable detection methods for diagnosis of HRVs infection. Currently, the detection of viral antigens by enzyme-linked immunosorbent assay (ELISA) [15], and detection of HRVs RNA by RT-qPCR [16, 17] are considered as the two most promising methods for timely diagnosis of patients who are at risk for HRVs infections. These methods have high sensitivity and specificity, but their implementation requires well-trained personnel, as well as expensive and technically complex instruments, and it would be difficult to use in a point-of-care setting. Therefore, the development of novel, rapid and sensitive detection strategies for the diagnosis of HRVs in critical ill patients is vital to treat the disease.

Another new molecular diagnostic technology based on isothermal amplification, reverse-transcription recombinase polymerase amplification (RT-RPA) [18, 19], has attracted much attention due to its low-cost, ease of use and high-accuracy [20]. Furthermore, RT-RPA can be performed under constant temperature in low-resource environments without the application of thermostable enzymes or complex thermal cycles. The isothermal amplification process was simple and efficient, and the targeted nucleic acid can be detected using visual detection within 20 min [21, 22]. Recently, RT-RPA has been widely exploited for the detection of various human pathogens with high-performance [23–26]. For instance, Koray et al. presented the efficient and accurate detection of COVID-19 detection using RT-RPA [25]. To date, there has not been any report on the detection of HRV-B species using RT-RPA and Cas12a.

Herein, we presented a rapid and sensitive RT-RPA-Cas12a assay to detect HRV-B by targeting the conservative sequence of capsid protein VP4 segment. The developed protocol is able to detect target RNA of 0.5 copies/µL within 35–45 min, enabling it easily applicable in a point-of-care setting.

## Materials and methods

### Clinical samples

Thirty nasopharyngeal aspirates were collected from individuals with symptoms of respiratory tract infections, and tested positive for HRV-A (5 clinical samples), HRV-B (20 clinical samples), or HRV-C-positive (5 clinical samples) based on the method, as previously described [27]. Ten other specimens were tested negative for HRV-B, including human metapneumovirus (HmPV), adenovirus (ADV), bocavirus (HBOV), and respiratory syncytial virus (RSV), and used as the negative control. All samples were obtained by Ningbo Municipal Center for Disease Control and Prevention (NCDC) between January, 2018 and December, 2020.

### Reagents

ssDNA FQ reporters were synthesized by GENEWIZ Inc. (Suzhou, China) via labeling with 6-FAM and BHQ1 at their 5′ and 3′ ends. Oligonucleotides for RT-RPA primers and in vitro synthesis of crRNA were manufactured by Sangon Biotech (Shanghai, China), and shown in Table 1. RT-RPA kit was purchased from Jiangsu lesun biotechnology Co., Ltd (Wuxi, China). Cas12a and its reaction buffer NEB buffer 2.1 were bought from New England Biolabs (MA, USA).

**Table 1:** Oligonucleotides employed for RT-RPA and crRNA in RT-RPA-Cas12a-mediated assay for rhinovirus species B detection

Assay	Name	Oligonucleotide sequences (5’-3’)
RT-RPA	HRV-B-F1	AYTAGTYTGGTCGATGAGGCT
	HRV-B-F2	AGCCTGCGTGGCGGCCARCCCAGC
	HRV-B-F3	ATGTGCTTGRTTGWGANTCCTCCGGCCCCTGAATG
	HRV-B-R1	ATATGCTGTGACCATAAGAMAA
	HRV-B-R2	CGGACACCCAAAGTAGTCGGTCCCRTCCCRGAATT
	HRV-B-R3	GAAACACGGACACCCAAAGTAGTCGGTCCCRTCCC
	HRV-B-R4	GTWGANACYTGHGCDCCCATGRTCACAGTATAT
RT-RPA-Cas12-based detection	HRV-B-crRNA1-F	GAAATTAATACGACTCACTATAGGGTAATTTCTACTAAGTGTAGATTCCTCCGGCCCCTGAATGCG
	HRV-B-crRNA1-R	CGCATTCAGGGGCCGGAGGAATCTACACTTAGTAGAAATTACCCTATAGTGAGTCGTATTAATTTC
	HRV-B-crRNA2-F	GAAATTAATACGACTCACTATAGGGTAATTTCTACTAAGTGTAGATACCGACTACTTTGGGTGTCC
	HRV-B-crRNA2-R	GGACACCCAAAGTAGTCGGTATCTACACTTAGTAGAAATTACCCTATAGTGAGTCGTATTAATTTC
	HRV-B-crRNA2r-F	GAAATTAATACGACTCACTATAGGGTAATTTCTACTAAGTGTAGATGGACACCCAAAGTAGTCGGT
	HRV-B-crRNA2r-R	ACCGACTACTTTGGGTGTCCATCTACACTTAGTAGAAATTACCCTATAGTGAGTCGTATTAATTTC
	HRV-B-crRNA3-F	GAAATTAATACGACTCACTATAGGGTAATTTCTACTAAGTGTAGATACTTTGGGTGTCCGTGTTTC
	HRV-B-crRNA3-R	GAAACACGGACACCCAAAGTATCTACACTTAGTAGAAATTACCCTATAGTGAGTCGTATTAATTTC
	HRV-B-crRNA3r-F	GAAATTAATACGACTCACTATAGGGTAATTTCTACTAAGTGTAGATGAAACACGGACACCCAAAGT
	HRV-B-crRNA3r-R	ACTTTGGGTGTCCGTGTTTCATCTACACTTAGTAGAAATTACCCTATAGTGAGTCGTATTAATTTC
	ssDNA-reporter-FQ	6-FAM-TTATTATT-BHQ1

### Production of standard RNA of the target region for HRV-B

**Fig. 1:**
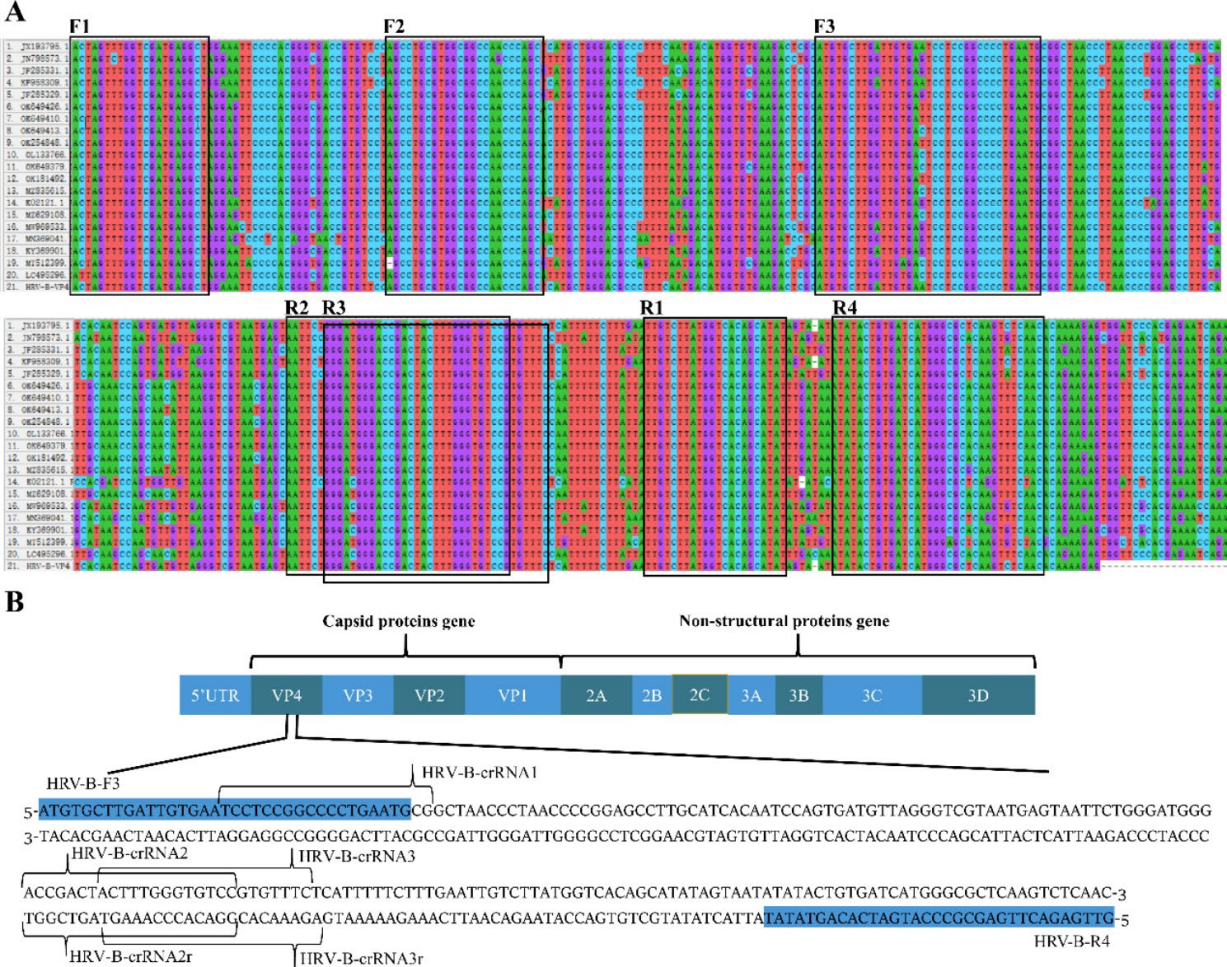
Schematic diagrams of RT-RPA-Cas12a-mediated fluorescence assay for the detection of human rhinovirus B (HRV-B). (**A**) Sequence alignment of available twenty whole genomes of HRV-B. The conserved nucleotide sequence in each position are presented by asterisks. Coloring of the nucleotide sequence was made according to the same pattern presented in MEGA7 software. (**B**) A schematic illustration different crRNA spacer sequence sites within the target VP4 gene sequence of HRV-B species genome. crRNA spacer sequences are indicated by blue colored text, respectively. RT-RPA: reverse-transcription recombinase polymerase amplification assay

Twenty whole genome sequences of HRV-B, including JX193795.1, JN798573.1, JF285331.1, KF958309.1, JF285329.1, OK649426.1, OK649410.1, OK649413.1, OK254848.1, OL133766.1, OK649379.1, OK181492.1, MZ835615.1, K02121.1, MZ629108.1, MW969533.1, MN369041.1, KY369901.1, MT512399.1, and LC495296.1, were retrieved from the National Center for Biotechnology Information and then aligned using the MEGA X. Comparative genomic analysis reveals that the segment of VP4 gene is highly conserved, and determined as the target region (Fig. 1A). Subsequently, the conserved fragment of VP4 gene composed of approximately 412 nucleotides was synthesized by Shanghai Sangon biotech (Shanghai, China), cloned into the pBluescript II SK (+) to manufacture pBluescript-VP4, and the recombinant plasmid was kept at -80 °C for further use.

For in vitro synthesis of standard RNA of the conserved target region for HRV-B, the *Sac* I-linearized pBluscript-VP4 was used as the template to biosynthesize standard RNA using the in vitro transcription (IVT) T7 Kit (TaKaRa, Dalian, China). Briefly, the IVT reaction mixture, including 5 µL of 10 × transcription buffer, 5 µL of each NTP solution (20 nM), 1.25 µL of RNase inhibitor (2 U/µL), 5 µL of T7 RNA polymerase (5 U/µL), 8.75 µL of RNase-free water and 10 µL of linear pBluscript-VP4 plasmid (100 ng/µL), was conducted at 42 ℃ for 2 h. Then, the number copies of standard RNA of the target region for HRV-B were determined using the method previously reported [24].

### Design of RT-RPA primer and crRNA

Based on the consensus sequences for HRV-B, both four pairs of RT-RPA primers and five pairs of oligonucleotides for preparation of crRNA were designed (Fig. 1B), followed by the synthesis by Suzhou GENEWIZ biotech, and shown in Table 1. The primer specificity was checked by nucleotide BLAST (blastn), and the secondary structure and dimer analysis were performed using Oligo Analyzer 3.1. Preparation of crRNA was mainly conducted using the following two-step. Six pairs of oligonucleotides were annealed to generate double strand DNAs (dsDNA), respectively, and then the resulting dsDNA products were transcribed by in vitro transcription using IVT T7 Kit. The in vitro transcription reaction was performed by incubating the mixture, including 5 µL of 10 × transcription buffer, 5 µL of each NTP solution (20 nM), 1.25 µL of RNase inhibitor (2 U/µL), 5 µL of T7 RNA polymerase (5 U/µL), 16.25 µL of RNase-free water and 2.5 µL of annealed dsDNA (100 ng/µL), at 42 ℃ for 2 h. The resulting crRNA products were subjected to phenol/chloroform extraction, and the amount of crRNA was measured using the spectrophotometer (Metash Instruments, Shanghai, China).

### Screening of optimal crRNA candidate for RT-RPA-Cas12a-mediated assay

To screen the optimal crRNA, five crRNA (crRNA1, crRNA2, crRNA2r, crRNA3 and crRNA3r) was designed, and their trans-cleavage efficiencies mediated by Cas12a were assessed by the intensity of fluorescent signal, respectively. Meanwhile, to examine the optimal concentration of crRNA for the trans-cleavage activities, final concentrations ranging from 0 nM to 120 nM were applied and assessed in the RT-RPA-Cas12a-mediated assay.

### RT-RPA-Cas12a-mediated assay

The RT-RPA-Cas12a-mediated assay is mainly divided into two steps. The first step of this assay involves the exponential amplification of the target sequence by RT-RPA. The 50 µL RT-RPA reaction mixture was performed in the preheated Axxin T8 isothermal instrument for 20 min at 39 ℃, including 25 µL of reaction buffer V, 1 µL of each primer (10 µM), 15 µL of ddH_2_O, 0.5 µL RNase inhibitor (40 U/µL), 5 µL of standard RNA (100 ng/µL) and 2.5 µL of 280 mM magnesium acetate. For the negative control, RNase-free water was served as the template in the same volume. At the second stage, the Cas12a-mediated trans-cleavage reaction was carried out in a 50 µL reaction mixture, including 5 µL of 10 × NEB Buffer 2.1, 2 µL of ssDNA FQ (1 µM), 1 µL of 2.5 µM Cas12a, 2 µL of 1 µM crRNA, 0.5 µL RNase inhibitor (40 U), 29.5 µL of deionized water, and 10 µL of RT-RPA products or deionized water (the negative control). The Cas12a-mediated reaction was performed at 37 °C for 30 min. The detection results of the assay were obtained with fluorescent signals collected every 20 s with ssDNA FQ as the substrates.

### Specificity and sensitivity of RT-RPA-Cas12a-mediated fluorescent assay

The specificity of RT-RPA-Cas12a-mediated fluorescent assay was performed by using RNA from several viral samples as templates, including HRV-B species, the mixture of human rhinoviruses A and C species (HRV-AC) in equal volume, HmPV, ADV, HBOV, and RSV, and the amount of template for per reaction was the final concentration of 200 copies/µL. 10 different samples tested positive for each test virus, and 10 HRV-AC samples were applied as the negative control.

To assess the sensitivity of RT-RPA-Cas12a-mediated fluorescent assay, a concentration gradient of standard RNA of the target region for HRV-B (1000, 100, 10, 1, 0.5, 0.1, 0.05 and 0 copies/µL), was used as the template. Each reaction was replicated three times and the results were examined using real-time fluorescence readout.

### RT-qPCR detection

The RT-qPCR assay for detection of HRV nucleic acids was performed using HRV kit (bioPerfectus technologies, Jiangsu, China) in an ABI 7500 (Applied Biosystems). Briefly, the total RNAs of 20 HRV-B samples in this study were extracted using automatic nucleic acid extraction instrument (bioPerfectus technologies, Jiangsu, China), respectively. The reaction was carried out in a 20 µL reaction mixture, including 10 µL of 2 × RT-qPCR buffer, 0.8 µL of Enzyme Mix, 0.4 µL of each primer (10 µM), 0.4 µL of ROX Dye II (10 µM), 5 µL of Total RNA, 3.4 µL of RNase-free ddH_2_O. The reactions were performed with first step of reverse transcription at 42 °C for 5 min, followed by 95 °C for 1 min, 40 cycles of 95 °C for 30 s, 60 °C for 30 s.

### Statistics

Data are representative of each sample with at least three independent biological replicates. Statistical analysis of fluorescence values was measured using the GraphPad Prism 8 (GraphPad Software, version 8.0.1), and statistical difference was calculated using the Students’ t-test.

## Results

### RT-RPA-Cas12a-mediated fluorescence assay for standard RNA of the target region for HRV-B

**Fig. 2:**
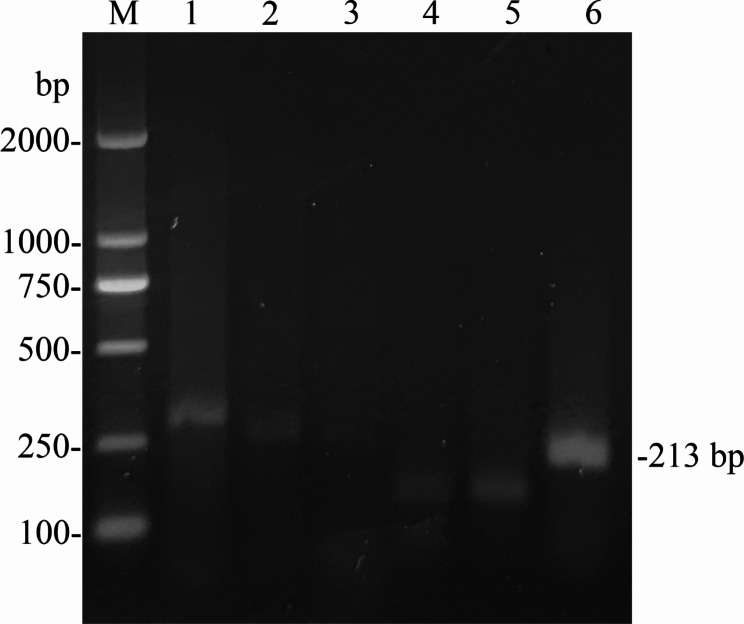
Screening of the optimal RT-RPA primers using agarose gel electrophoresis. M, DL2000 DNA marker; 1–6, RT-RPA products were amplified using the combination of different primers, including HRV-B-F1/HRV-B-R1, HRV-B-F1/HRV-B-R2, HRV-B-F2/HRV-B-R1, HRV-B-F3/HRV-B-R2, HRV-B-F3/HRV-B-R3, and HRV-B-F3/HRV-B-R4, respectively

The primer pair and crRNA sequences for RT-RPA-Cas12a-mediated fluorescence assay were optimized. Firstly, the performance of five pairs of primers was examined using RT-RPA assays with the final concentration of 1000 copies/µL of standard RNA of the target region for HRV-B as the template. RT-RPA products of different primer pairs were analyzed using agarose gel electrophoresis. As shown in Fig. 2, HRV-B-F3/HRV-B-R4 was capable of producing a clear single and expected 213 bp DNA band, and was selected for subsequent RT-RPA experiments.

**Fig. 3:**
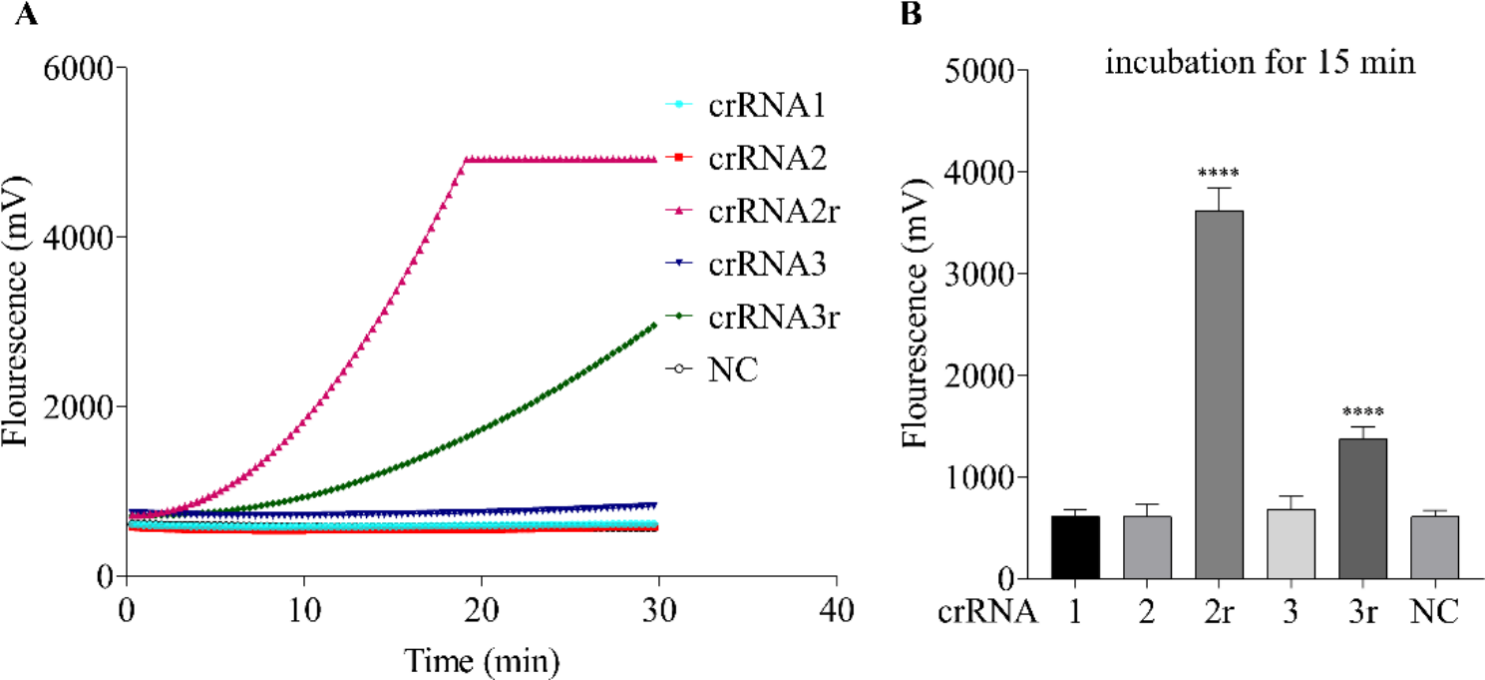
Evaluation the performance of different crRNA sequence in RT-RPA-Cas12a-mediated fluorescence assay with ssDNA FQ. (**A**) Representative real-time fluorescence kinetics of single crRNA sequence in the RT-RPA-Cas12a-mediated fluorescence assay. (**B**) Fluorescence values of single crRNA sequence obtained at 15–25 min. The data were shown as mean ± SD in each group with at least three independent experiments. NC, negative control group

Besides, the crRNA sequence was also optimized, because the stronger fluorescence signal was an important parameter for the ideal detection method. To evaluate the effect of crRNA sequences on detection efficiency and sensitivity, the efficiency mediated by different crRNA in the RT-RPA-Cas12a-mediated fluorescence assay was evaluated. In this assay, final concentrations of each crRNA and Cas 12a were 40 nM and 50 nM, respectively. As presented in Fig. 3, the results indicate that only the crRNA2r and crRNA3r sequence could form a ternary complex with Cas12a and target sequence, and produced detectable fluorescence signals. In contrast, no obvious fluorescence signals were observed for poorly-performing crRNA1, crRNA2 and crRNA3. Particularly, the strongest fluorescence signal was observed for the crRNA2r sequence, and reached the reaction plateau over the following 20 min. Moreover, the end-point fluorescence values mediated by various crRNA sequences were shown in Fig. 3B, the results also show that crRNA2r generated the highest fluorescence intensity compared with other crRNA treated group (*p* < 0.001). Thus, crRNA2r was selected for subsequent Cas12a detection reaction.

### Specificity of the RT-RPA-Cas12a-mediated fluorescence assay

**Fig. 4:**
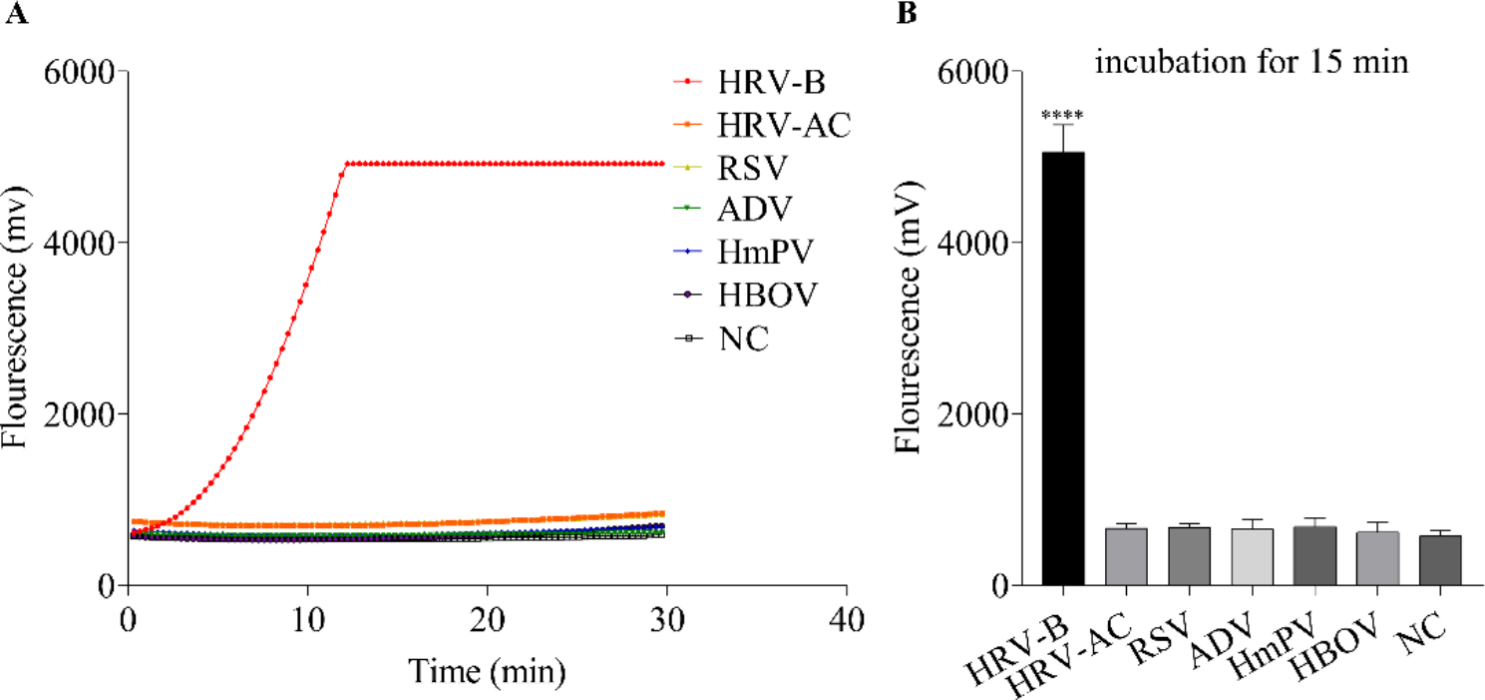
Specificity evaluation of RT-RPA-Cas12a-mediated fluorescence assay using RNA from viral samples, including human rhinoviruses B (HRV-B) species, the mixture of human rhinoviruses A and C species (HRV-AC) in equal volume, metapneumovirus (HmPV), adenovirus (ADV), bocavirus (HBOV), and respiratory syncytial virus (RSV), as the template of RT-RPA, respectively. (**A**) Specificity evaluation of RT-RPA-Cas12a-mediated real-time fluorescence assay with a fluorescence detector readout. (**B**) Specificity examination of the RPA-Cas12a-mediated end-point fluorescence assay at 15 min. The data are presented as mean ± SD (n = 3). NC, negative control group

The specificity and feasibility of RT-RPA-Cas12a-mediated fluorescence assay were tested for different samples, including standard RNA of HRV-B, HmPV, ADV, HBOV, RSV, and HRV-AC. As shown in Fig. 4A, in the RT-RPA-Cas12a-mediated real-time fluorescence assay, only standard RNA of HRV-B was found to produce remarkable fluorescence signals in the detection samples, compared to other samples, and no cross-reactivity was observed for other viral samples with the final concentration of 200 copies/µL. To obtain the quantitative data of the RT-RPA-Cas12a-mediated fluorescence assay, the end-point fluorescence values at 15 min were calculated. As shown in Fig. 4B, the significant difference of fluorescence values was observed only between standard RNA-added group and the negative group (*p* < 0.001). These results indicate that the RT-RPA-Cas12a-mediated fluorescence assay for detection of HRV-B exhibited the robust specificity.

### Sensitivity of the RT-RPA-Cas12a-based fluorescence assay

**Fig. 5:**
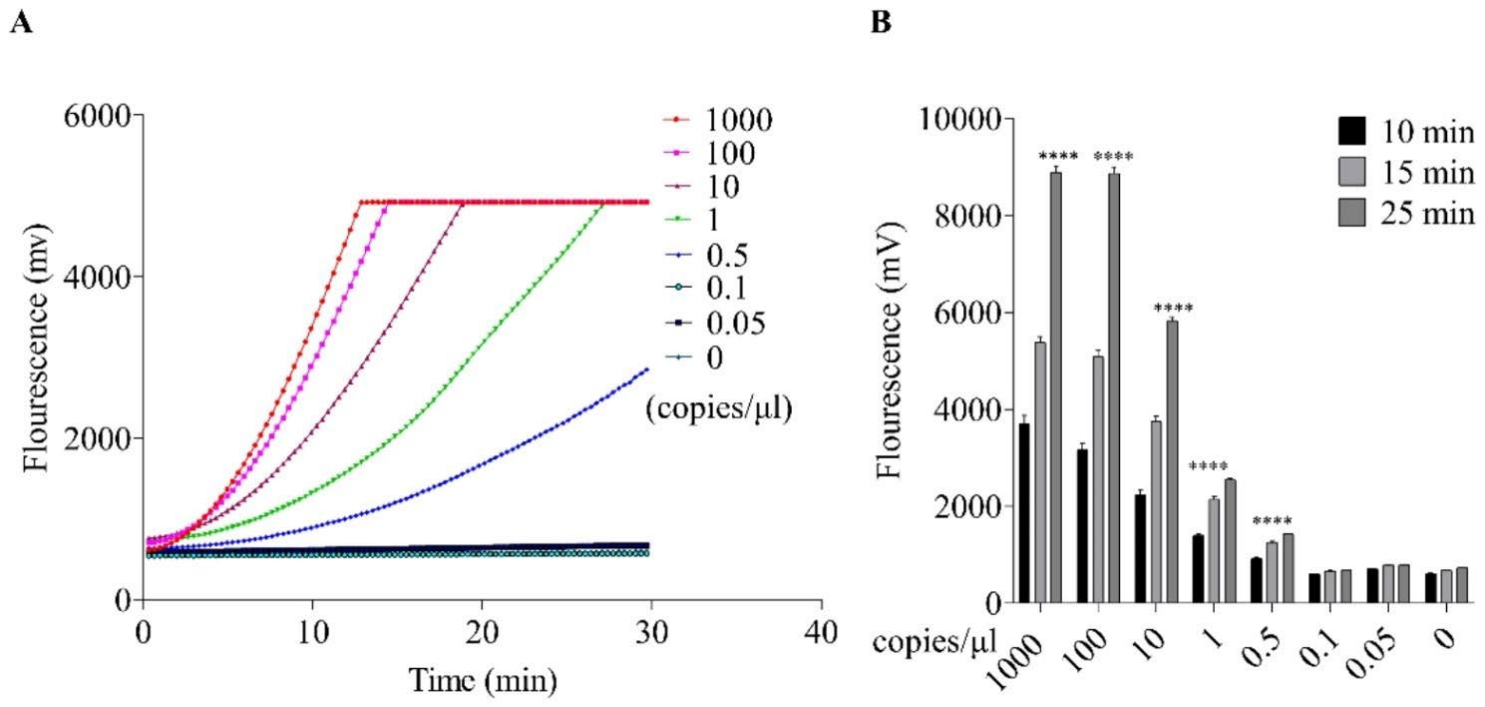
Examination of the sensitivity of the RT-RPA-Cas12a-mediated fluorescence assay using a serial dilution of the standard RNA of human rhinovirus B (HRV-B) as the template. (**A**) Sensitivity examination of RT-RPA-Cas12a-mediated real-time fluorescence assay for HRV-B. (**B**) Sensitivity evaluation of RT-RPA-Cas12a-mediated end-point fluorescence assay at different times. The values were presented as the means ± SD in each group with at least three independent experiments. NC, negative control group

The sensitivity of the RT-RPA-Cas12a-based fluorescence assay was evaluated using different gradient concentrations of target standard RNA, including 0, 0.05, 0.1, 0.5, 1, 10, 100, and 1000 copies/µL. As shown in Fig. 5A, in the RT-RPA-Cas12a-based real-time fluorescence assay, fluorescence values produced by the Cas12a-mediated trans-cleavage activities were proportional to serial dilutions of target standard RNA. An obvious fluorescent signal was observed when the final concentration of target standard RNA was more than 0.5 copies/µL. Moreover, Fig. 5B shows that in the RT-RPA-Cas12a-mediated end-point fluorescence assay, 0.5 copies/µL of target standard RNA produced a significant difference of fluorescence values compared with the negative control when the trans-cleavage for 25 min was optimum. The results showed that the minimum RNA amount in RT-RPA-Cas12a-mediated fluorescence assay was 2.5 copies of substrate target RNA per reaction.

### Clinical examination of the RT-RPA-Cas12a-mediated fluorescence assay

To examine whether RT-RPA-Cas12a-mediated fluorescence assay can be used for monitoring of virus infections, the HRV-B detection was conducted for clinical samples. 30 samples were collected, and total RNA was manually extracted using magnetic bead-based extraction kit combined with a simple magnetic separator, respectively. Then, the extracted RNA samples were tested using both the RT-RPA-Cas12a-mediated fluorescence assay and RT-qPCR following the above procedure, respectively. As demonstrated in Table 2, the positive predictive agreement between RT-RPA-Cas12a-mediated fluorescence assay for HRV-B samples with RT-qPCR assay were 90%, whereas the negative predictive agreement was 100%. These results revealed that a robust diagnosis concordance of our RT-RPA-Cas12a-mediated fluorescence assay and RT-qPCR was observed.

**Table 2:** Comparison of RT-RPA-Cas12a-mediated fluorescence assay and qRT-PCR of rhinovirus species B for 30 samples

Assay	Number of samples		Coincidence rate with qRT-PCR for 20 positive samples	Coincidence rate with qRT-PCR for 10 negative samples
	Positive	Negative		
RT-RPA-Cas12a-mediated fluorescence	18	10	90%	100%
qRT-PCR	20	10		

## Discussion

HRVs are a major contributor of acute exacerbations of chronic obstructive pulmonary disease and asthma exacerbations in children, and is a significant problem for healthcare systems around the world [1, 28, 29]. More than 150 serotypes of HRVs have been defined, and classified into three different groups: HRV-A, HRV-B, and HRV-C [30]. The two species HRV-A and HRV-B were originally discovered in the 1950s, while the HRV-C species were identified in 2006, included in the International Committee on Taxonomy of Viruses in 2009 [31]. A previous study reported that HRV-A was common all year round, whereas HRV-C was more common in winter, and HRV-B exhibited an unclear seasonality [32]. In general, the primary routes of HRV transmission are attributed to hand direct contact between indivdualss or indirect contact through fomites [33], which can in turn increase the risk of the rapid spread of HRVs in public settings. The resulting remarkable economic and health burden associated with HRVs infection underlines the need to exploit a rapid and sensitive method for accurate and timely diagnosis of HRVs infection among asymptomatic or mildly symptomatic individuals, which can enhance timeliness and precision of anti-infective treatment and interrupt transmission of infection. Currently, RT-qPCR is the most widely used laboratory method to detect HRVs infections [17, 34]; nevertheless, RT-qPCR is unsuited for fast and POCT detection for HRVs in resource-limited setting due to the need of expensive equipment and skilled technicians. Thus, it is essential to exploit a novel, rapid and simple method for detecting HRV-B infection based on isothermal nucleic acid-based amplification technology. Recently, the combination of isothermal nucleic acids amplification and clustered regularly interspaced short palindromic repeats (CRISPR) systems has been extensively exploited in nucleic acid detection duo to the advantages of simple amplification, high sensitivity and high specificity [23, 35]. Especially, the CRISPR-Cas12a system can trans cleave single-stranded DNA and can specifically realize the detection of DNA target [24].

In this study, to exploit the RT-RPA-Cas12a-mediated fluorescence assay to diagnose HRV-B infection, the target sequence of VP4 region of HRV-B species was determined. Then, the RT-RPA primer and crRNA that designed based on the conserved VP4 gene sequence of HRV-B was screened. The combination of double targeting of RT-RPA primer and crRNA could improve the specificity of the detection of HRV-B with minimal off-target background noise. Significantly, in this study, five crRNA sequences with or without a specific protospacer adjacent motif (PAM), including crRNA1, crRNA2, crRNA2r, crRNA3 and crRNA3r, were designed. The results indicate the trans-cleavage activity of CRISPR/Cas12a was induced by crRNA2r and crRNA3r sequence, which would dramatically expand the application of CRISPR/Cas12a in the diagnosis of infectious pathogens [35, 36]. However, this process involves intensive manual design and stringent evaluation for experiment to obtain active crRNA. Moreover, the detection of HRV-B can be completed within 35–45 min, including 20 min of target sequence amplification and 15–25 min of trans-cleavage reaction, which was faster compared with the RT-qPCR method. Meanwhile, our RT-RPA-Cas12a-mediated fluorescence assay obtained a lower detection threshold with 2.5 copies of target RNA per reaction (0.5 copies/µL). To evaluate the utility of our RT-RPA-Cas12a-mediated fluorescence assay on clinical specimens, manual RNA extractions of 30 human respiratory specimens were tested. And the established RT-RPA-Cas12a-mediated assay achieved highly consistent results with that of RT-qPCR method, with 90% positive predictive agreement and 100% negative predictive agreement for HRV-B.

In conclusion, we developed a RT-RPA amplification with CRISPR/Cas12a detection system for the rapid detection of HRV-B with a detection threshold of 0.5 copies/µL. To our knowledge, this is the first application for monitoring HRV-B using the RT-RPA-CRISPR technique, which is a promising tool for on-site detection method for HRV-B infections in resource-poor or constrained settings.

## Data Availability

All data generated or analyzed herein are included in this manuscript.
